# Removal of Cadmium from Aqueous Solutions by *Saccharomyces cerevisiae–*Alginate System

**DOI:** 10.3390/ma12244128

**Published:** 2019-12-10

**Authors:** Silvia Carolina Moreno Rivas, Rosa Idalia Armenta Corral, María del Carmen Frasquillo Félix, Alma Rosa Islas Rubio, Luz Vázquez Moreno, Gabriela Ramos-Clamont Montfort

**Affiliations:** Centro de Investigación en Alimentación y Desarrollo, A.C. Carretera Gustavo Enrique Astiazarán No. 46, Col. La Victoria, C.P. 83304 Hermosillo, Sonora, Mexico; carolina.moreno@estudiantes.ciad.mx (S.C.M.R.); rosy@ciad.mx (R.I.A.C.); cfrasquillo@ciad.mx (M.d.C.F.F.) aislas@ciad.mx (A.R.I.R.); lvazquez@ciad.mx (L.V.M.)

**Keywords:** cadmium, yeast, immobilization, biosorbent, drinking water

## Abstract

The aim of this study was to determine the Cd^2+^ removal capacity of a biosorbent system formed by *Saccharomyces cerevisiae* in calcium alginate beads. The adsorption of Cd^2+^ by a *S. cerevisiae*–alginate system was tested either by batch or fixed-bed column experiments. The *S. cerevisiae*–alginate system was characterized using dynamic light scattering (DLS, zeta potential), size, hardness, scanning electron microscopy (SEM), and Fourier-transform infrared spectroscopy. Beads of the *S. cerevisiae*–alginate system showed a spherical–elliptical morphology, diameter of 1.62 ± 0.02 mm, 96% moisture, negative surface charge (−29.3 ± 2.57 mV), and texture stability during storage at 4 °C for 20 days. In batch conditions, the system adsorbed 4.3 µg of Cd^2+^/g of yeast–alginate beads, using a Cd^2+^ initial concentration of 5 mg/L. Adsorption capacity increased to 15.4 µg/g in a fixed-bed column system, removing 83% of total Cd^2+^. In conclusion, the yeast–alginate system is an efficient option for the removal of cadmium at low concentrations in drinking water.

## 1. Introduction

Cadmium (Cd^2+^) is an extremely toxic and environmental persistent heavy metal. Long-term exposure to low concentrations of Cd^2+^ may imply chronic intoxication strongly associated with irreversible damage in the liver, kidneys, lungs, bones, and reproductive system [1]. In addition, cadmium and cadmium-containing substances are classified as carcinogens by regulatory agencies in the world [2,3,4].

Cadmium may pollute the aquatic environment from a variety of industrial water discharges that include electroplating, pigments, and battery production wastes, as well as process waters from smelters, and iron and steel plants. However, the largest contributors to cadmium water contamination are fertilizers produced from phosphate ores, concentrate processing waters from mines, and leakages from mine tailings [2,5]. Cadmium may also be found in drinking water supplies as a result of damage and deterioration of galvanized plumbing systems [2,6]. Therefore, in order to reduce Cd^2+^ exposure in the general population, control of the discharge of contaminants to the environment, management and treatment of wastewaters from the metallurgic industry should be applied [7].

Also, freshwater bodies polluted with low concentrations of Cd^2+^ need to be treated in order to reduce the amount of metal to levels under maximum permissible limits of 0.005 mg/L. Different processes, including the use of conventional adsorbents, chemical precipitation, ion exchange, coagulation/filtration, and membrane separation are applied for the treatment of Cd^2+^-bearing industrial effluents [5]. However, these methods are expensive or produce a large volume of sludge when treating water containing Cd^2+^ in low concentration (1–100 mg/L). Also, these treatments can consume large quantities of chemical products or high amounts of energy [8,9]. Therefore, low-cost and efficient alternatives are needed. In this context, biosorption is one of the most promising decontamination methods [10].

The use of any metal biosorbent is an environmentally friendly option. Moreover, metal biosorbents are cheaper than other conventional methods, and they are more versatile and diverse in terms of their molecular composition due to the variety of sources from which they can be obtained [11]. Biosorption includes the use of generally recognized as safe (GRAS) polymers (e.g., alginate, chitosan) and microbial biomass such as lactic acid bacteria and yeast [12,13]. Treatment of cadmium pollution by microbial action is a promising alternative due to its capacity to remove metal in water and food, either via active or passive uptake, which includes processes like microprecipitation, ion exchange, physical adsorption, coordination, and complexation [14,15,16].

The yeast *S. cerevisiae* is widely used in food biotechnology processes, like bread, wine, and beer production. It is a GRAS organism. For this reason, efficient and low-cost processes were developed to produce large-scale yeast biomass. In addition, *S. cerevisiae* biomass can also be obtained as a high-volume brewing by-product. *S. cerevisiae* is a promising biosorbent that shows the capacity to remove heavy metals from aqueous solutions, such as lead, cadmium, chromium, copper, nickel, and zinc, even when inactive, and it shows good efficiencies in short contact times [17,18,19].

However, after biosorption treatment, the cell–liquid separation may be a problem, adding a unit operation to the process. Therefore, the immobilization of yeast cells in a fixed matrix is an alternative for their application [20,21]. Alginate is an easily available, biocompatible, and gel-forming linear polysaccharide obtained from brown seaweeds [22]. Calcium (Ca^2+^) and other divalent cations induce the covalent crosslinking of alginate; therefore, gel formation under simple and mild conditions makes it possible to immobilize eukaryotic cells [23]. In addition, empty calcium alginate beads are used as adsorbent for the recovery of heavy metals from aqueous solutions [24]. Therefore, the encapsulation of biosorbent microorganisms in a biopolymer such as alginate could result in a more efficient biosorption system which combines the potential of both components, in addition to being a more stable, easy-to-handle, economical, and safe solution [25]. *Saccharomyces cerevisiae* could be a feasible option. This strategy would provide an eco-friendly Cd^2+^ removal system, mainly from water with low metal ion concentration, which may not be feasible using conventional methods. The aim of this study was to determine the Cd^2+^ removal capacity of a biosorbent system formed by *S. cerevisiae* immobilized in a calcium alginate gel matrix for its potential application in drinking water. The yeast–alginate system was also characterized using different techniques.

## 2. Materials and Methods

### 2.1. Materials and Microorganism

Low-viscosity sodium alginate (130–300 mPa) was purchased from BÜCHI (Labortechnik, Flawil, Switzerland). Cadmium standards were acquired from Sigma-Aldrich (St Louis, MI, USA). All other chemicals were analytical grade from Sigma-Aldrich (St Louis, MI, USA), unless otherwise specified.

Dried commercial *Saccharomyces cerevisiae* (Magidely^®^, Mexico) was obtained from three local markets from Hermosillo, Mexico. Yeast biomass was inactivated by heating in an autoclave for 30 min at 121 °C, then frozen at −40 °C and lyophilized for 48 h (VirTis Benchtop 3.3, Warminster, PA, USA). The lyophilized cells were stored in a freezer at −40 °C until encapsulation.

### 2.2. Cadmium Solutions

A stock solution of 100 mg/L was obtained from a standard Cd^2+^ solution for atomic absorption of 1000 mg/L (Sigma-Aldrich, St. Louis, MI, USA). Two working solutions were prepared by dilution with ultrapure water at concentrations of 5 and 10 mg/L.

### 2.3. Synthesis of the *S. cerevisiae*–Alginate System

*S. cerevisiae*’s encapsulation was carried out using an external ionic gelation technique with a semi-automatic Encapsulator B-395 equipped with a 750-µm nozzle (BUCHI, Labortechnik, Flawil, Switzerland). The vibration frequency was adjusted to 350 Hz to generate 350 droplets per second. Yeast biomass (1.5 g/L) was dispersed in a 1.5% sodium alginate solution. The mixture was dispensed into an inlet reservoir of an airflow-assisted nozzle system and applied dropwise (200 mBar, 1100 V) into a 0.4 M CaCl_2_ crosslinking solution under gentle stirring (70 rpm) with a magnetic bar. Synthetized beads were maintained in the crosslinking solution for 30 min to complete the hardening process, recovered with a micro-sieve, and washed three times with deionized water to remove CaCl_2_ excess. Produced beads (eight batches) were routinely stored at 4 °C for further experiments. Empty Ca-alginate beads (without yeast) were used as a control.

### 2.4. Characterization and Microstructural Analysis

The obtained *S. cerevisiae*–alginate system was characterized and analyzed based on particle size distribution, optical microscopy, microstructural analysis, surface charge, and moisture content.

#### 2.4.1. Particle Size Distribution

Wet *S. cerevisiae* alginate beads were subjected to size analysis using an electronic digital caliper (Traceable^®^, Control Company, Webster, TX, USA), with a diameter measurement of 150 beads. In addition, size distribution analyses were performed with an inverted microscope Zeiss Axio Vert A1 (Karl-Zeiss, Göttingen, Germany). The diameters of 20 arbitrarily chosen beads were measured using Zen 2 Lite software (Karl-Zeiss, Göttingen, Germany). Measurements for control and treatments were performed in duplicate.

#### 2.4.2. Optical Microscopy Characterization

Morphology of wet alginate beads loaded with *S. cerevisiae* was characterized with a Stemi DV4 stereomicroscope (Karl-Zeiss, Göttingen, Germany). Beads were placed in a petri dish containing deionized water and gently shaken for better separation. Optical images were obtained using a digital camera (Pentax K500, Pentax, Tokyo, JPN).

#### 2.4.3. Microstructural Analysis

Microstructure of lyophilized alginate beads was characterized by scanning electron microscopy (SEM) using a JEOL-JSM-5900 LV microscope (JEOL USA Inc, Peabody, MA, USA), operating at 10 kV electron acceleration and 100 mm of focal length. Samples were coated with a thin gold layer (60 A) prior to observation. Images of empty Ca-alginate control and *S. cerevisiae*–alginate system beads were obtained before and after incubation (15 min) in a water solution containing 5 mg/L Cd^2+^. Particle size of lyophilized alginate beads was determined by SEM microscopy.

#### 2.4.4. Surface Charge and Moisture Content

The surface charge was obtained by triplicate measurements of zeta potential (Zetasizer Nano-ZS90, Malvern Instruments Ltd., Worcestershire, UK) using deionized water as a diluent. The moisture content of capsules was determined using the AOAC 4.1.06 method [26] in triplicate.

### 2.5. Texture Analysis during Storage

The mechanical resistance of beads was determined in order to observe the performance of beads firmness during storage (for possible preservation). Firmness measurements were carried out at days zero, four, eight, 12, 16, and 20 of storage in refrigeration (5 °C) in deionized water. Calcium alginate beads without yeast were used as control. A texturometer (Texture Analyzer, Model TA-TX2, Stable Micro Systems Ltd., Godalming, UK) was used with a load cell of 5 kg and a semi-cylindrical acrylic probe of 18 mm of diameter and 39 mm of height. Beads were placed on a flat stainless-steel surface underneath the probe, removing water excess with a soft tissue. A compression of 40% was applied to each bead with a test speed of 0.1 mm/s. A force (gf) versus time (s) plot was obtained and the max force value (gf) was recorded as bead firmness. At least 15 measurements were performed for each treatment.

### 2.6. Batch Biosorption of Cd^2+^ by *S. cerevisiae*–Alginate System

For batch biosorption experiments, 20 mL of 5 mg/L or 10 mg/L Cd^2+^ working solutions at pH 6.0 were individually mixed with 12 g of calcium alginate beads loaded with *S. cerevisiae* in 50-mL conical tubes. These conditions were chosen because they proved to be the most effective for the biosorption of cadmium by yeast [27]. After 5, 15, 30, or 60 min of incubation at 25 °C, beads separated by gravity. The supernatant was recovered and acidified with 80 μL of ultrapure HNO_3_ for the analysis of non-adsorbed residual Cd^2+^ by atomic absorption spectroscopy (AAS) (Agilent Technologies 240FS-AA, Santa Clara, CA, USA). Controls included free *S. cerevisiae* cells, Cd^2+^ solutions without biosorbent, and empty Ca-alginate beads. Free cells of *S. cerevisiae* were separated from supernatant by centrifugation at 4500 rpm for 10 min at 15 °C for non-adsorbed residual Cd^2+^ determination.

Cadmium removal efficiency and adsorption capacity were calculated using the following equations:

Removal efficiency (%) = ((C_i_ − C_t_)/C_i_) × 100%, (1)

q_t_ = ((C_i_ − C_t_)V)/m, (2)

where q_t_ is the adsorption capacity at time t (µg/g), C_i_ is the initial concentration of Cd^2+^ (µg/mL), C_t_ is the Cd^2+^ concentration at time t (µg/mL), V is the volume of the solution (mL), and m is the mass of the adsorbent (g).

### 2.7. Fixed-Bed Column Biosorption

For fixed-bed column biosorption, a glass column (height 12.2 cm, inner diameter 2.8 cm) was packed with 12 g of calcium alginate beads loaded with *S. cerevisiae*. The temperature of the column was maintained at 25 °C throughout all experiments. Fifteen milliliters of Cd^2+^ aqueous solution (5 mg/L, pH 6.0) was pumped through the column (1.5 mL/min, 15 min resident time) in an up-flow mode with a peristaltic pump (Bio-Rad, Hercules, CA, USA). After resident time, samples were collected, acidified, and analyzed for residual Cd^2+^ as described before. For desorption experiments, 50 mL of 25% HNO_3_ was applied (1.5 mL/min, 50 min resident time) to the packed beads [28]. The amount of total Cd^2+^ desorbed was determined by AAS from the filtered eluates. Following elution, beads were washed twice with 50 mL of deionized water.

### 2.8. Fourier-Transform Infrared (FTIR) Spectroscopy

FTIR spectra of the *S. cerevisiae*–alginate system were recorded before and after Cd^2+^ adsorption. Empty Ca-alginate beads and free *S. cerevisiae* were used as controls. Samples were prepared as discs using potassium bromide (KBr). FTIR spectra were obtained in a NICOLET Protégé 460 (Cole Parmer, Vernon Hills, IL, USA) spectrometer with WinFIRST version 2.10 software (Analytical Technology, Inc., Madison, WI, USA). The spectrum was recorded in the range of 4000 to 400 cm^−1^. Spectra were normalized at the 1400 cm^−1^ region using Origin Pro 9 software (OriginLab Corporation, Northampton, MA, USA).

### 2.9. Statistical Analysis

Results were expressed as means ± standard error. Values were treated by analysis of variance (ANOVA) and Tukey–Kramer test to determine significant differences between means (*p* ≤ 0.05). Minitab 17 (LEAD Technologies, Charlotte, NC, USA) statistical software and SigmaPlot 11 (Systat Software, Chicago, IL, USA) were used for data analysis and graphic representations, respectively.

## 3. Results and Discussion

### 3.1. Characterization and Microstructural Analysis

**Figure 1:**
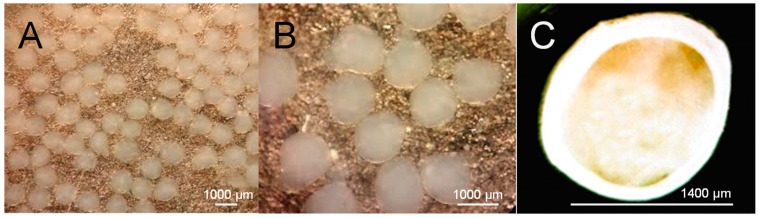
Morphology of *Saccharomyces cerevisiae*–alginate bead system. (**A**) Stereo microscope image (8×); (**B**) stereo microscope image (16×); (**C**) inverted microscope image.

Empty Ca-alginate beads showed almost a spherical–elliptical morphology with a continuous smooth surface and a diameter of 1.51 ± 0.02 mm (Figure 1). Morphology of *S. cerevisiae*-loaded beads was similar to empty Ca-alginate beads. However, the diameter was significantly (*p* ≤ 0.05) larger (1.62 ± 0.02 mm). These results are in agreement with earlier reports for microorganism encapsulation generated by semi-automated vibration technology [29,30].

**Figure 2:**
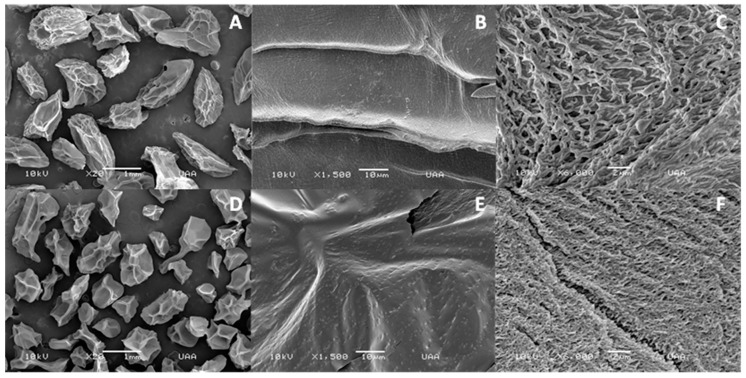
Scanning electron microscopy (SEM) images of the outer surface of empty Ca-alginate beads (**A**–**C**) before exposure to Cd^2+^, and (**D**–**F**) after Cd^2+^ exposure.

**Figure 3:**
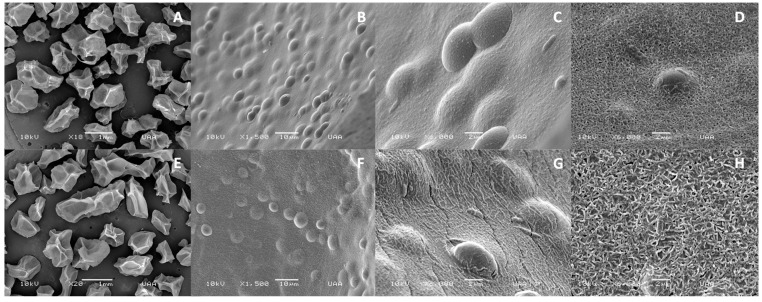
Scanning electron microscopy (SEM) images of the outer surface of *Saccharomyces cerevisiae*–alginate system (**A**–**D**) before exposure to Cd^2+^, and (**E**–**H**) after Cd^2+^ exposure.

SEM images of the lyophilized Ca-alginate beads, as well as their shapes and surfaces, are shown in Figure 2A and Figure 3A. Beads were amorphous with cavities or indentations that may be attributed to shrinkage and partial collapse of the alginate gel network during lyophilization [31]. The mean particle size of lyophilized beads was 1.49 ± 0.07 and 1.37 ± 0.05 mm for empty and yeast-loaded beads, respectively (Table 1). Lyophilization had no significant effect on the size of empty Ca-alginate beads (*p* > 0.05); however, a reduction in the size of lyophilized capsules loaded with yeast was observed, compared to the freshly synthesized ones (*p* ≤ 0.05). This was attributed to a lower polymer concentration inside the bead than the surface, related to cell incorporation, which led to a greater shrinkage after water loss by freeze-drying [32].

Incubation with Cd^2+^ produced a bead size reduction which was significant (*p* ≤ 0.05) in the empty ones (Table 1). This could be attributed to a major interaction between Cd^2+^ ions and mannuronic regions of empty Ca-alginate beads that could impact the capsule structure [24]. This size reduction was less evident in the yeast-loaded alginate beads, which may indicate that the presence of yeast provides a greater stability to bead structure because of the formation of cell clusters on the bead surface [33].

Surface morphology of calcium alginate beads was analyzed before and after exposure to cadmium (Figure 2A–F). The surface of unexposed beads was more uniform and smoother (Figure 2B) than the exposed ones, which showed particle deposition (Figure 2E), small cavities, and surface splits (Figure 2F). These changes were observed in other biosorbent studies and appear due to the presence of heavy metals at the surface, after the biosorption process [34,35]. In addition, a significant reduction in the pore size was observed in empty Ca-alginate beads after Cd^2+^ contact (Table 1). This behavior could be explained by the fact that the alginate structure is freely exposed to Cd^2+^, without the physical barrier of yeast, with greater interactions with mannuronic sites that impact bead structure [36,37,38].

Scanning electron micrographs confirmed the effective immobilization of *S. cerevisiae* into alginate beads. Alginate-coated yeast cells were evidenced on the bead surface as seen in Figure 3B,C. Beads were also analyzed by SEM microscopy after Cd^2+^ exposure. This process changed the particle and pore size (Table 1). Surface morphology of the particles also changed. Reticulated patterns appeared in the surface of the bead, probably due to the cadmium adsorption to alginate [39]. Panda and Sarkar reported a significant increase in surface roughness produced by the interaction of chromium ions with alginate [40].

The zeta potential of all alginate beads was negative (Table 1) being greater (*p* ≤ 0.05) for beads loaded with *S. cerevisiae* (−26.9 to −32.0 mV) than for empty beads (−12.2 to −19.7 mV). The negative charge of empty Ca-alginate beads is mainly attributed to the carboxyl groups of β-d-mannuronic acid units of the polymeric alginate structure due to most of the α-l-guluronic acid units being crosslinked with calcium ions forming the hydrogel structure [41]. On the other hand, the zeta potential of *S. cerevisiae* is attributed to negatively charged functional groups, like phosphates and carboxylic groups, from cell-wall proteins and polysaccharides [42].

A negative biosorbent system surface charge favors the electrostatic interaction with Cd^2+^ and, thus, its removal from aqueous solutions [13,43]. Cadmium-enriched water exposition shifted the surface charge of all alginate beads from negative to positive zeta potentials (Table 1), ranging from 4.9 to 9.9 mV for empty Ca-alginate beads and 1.7 to 5.1 mV for alginate beads loaded with *S. cerevisiae*. These results indicate that the Cd^2+^ cations formed a secondary shell around the anionic calcium alginate beads.

### 3.2. Texture Analysis during Storage

**Figure 4:**
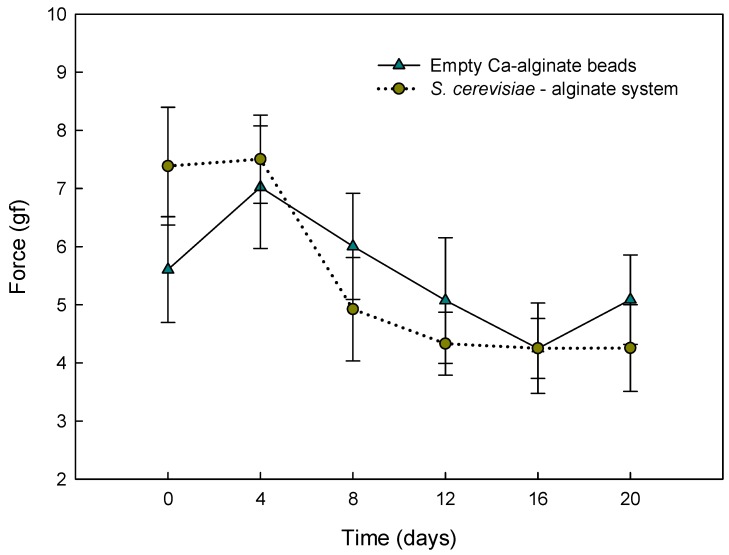
Texture stability of *S. cerevisiae*–alginate system and empty Ca-alginate beads after 20 days of storage at 4 °C.

The effect of storage time on the gel force of alginate beads was studied over 20 days (Figure 4). Texture changes were similar (*p* > 0.05) between empty Ca-alginate beads and *S. cerevisiae*-loaded alginate beads. The general behavior in both beads was an apparent gradual decrease in alginate gel strength over storage time. This loss of strength was higher after eight days of storage in both types of beads (6.0 ± 0.9 gf, 4.9 ± 0.9 gf), without significant changes in the subsequent days (*p* > 0.05). The observed diminution may be attributed to an ion exchange process between calcium and some residual cations in solution, like sodium and magnesium, and also to a slow dissociation of calcium to aqueous media [44,45,46].

### 3.3. Batch Biosorption of Cd^2+^ by *S. cerevisiae*–Alginate System

**Figure 5:**
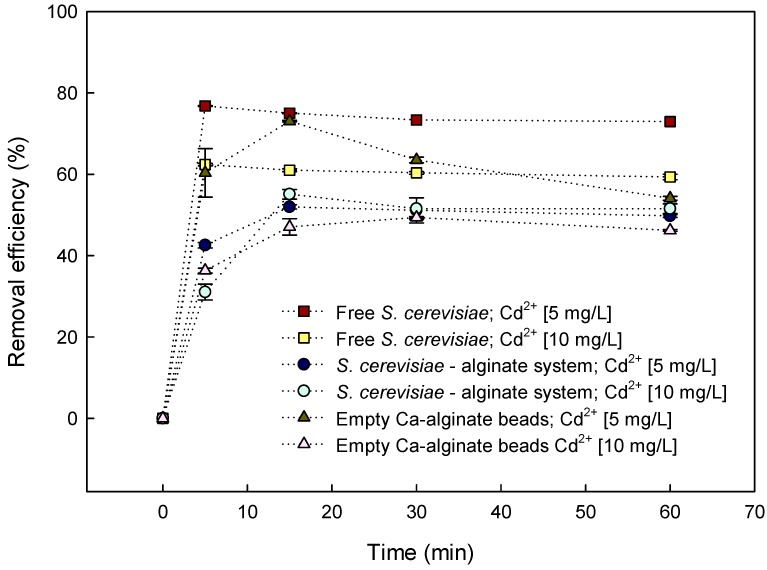
Effect of contact time on Cd^2+^ removal efficiency of free *S. cerevisiae* and *S. cerevisiae*–alginate system and empty Ca-alginate beads at 25° C, pH 6.0, and different initial Cd^2+^ concentrations.

*S. cerevisiae* binds heavy-metal cations on its outer surface due to the presence of negatively charged phosphomannan, phosphate, and carboxyl groups [9]. Batch biosorption studies showed that encapsulated *S. cerevisiae* was less effective in removing cadmium in solution at both concentrations (5 mg/L and 10 mg/L) than its free form (Figure 5). This effect was mainly due to a reduction of contact surface, which led to less exposure of functional groups to interact with Cd^2+^ ions. Also, despite the fact that encapsulation of yeasts by alginate provided a greater number of carboxyl groups, the increase in granular size of *S. cerevisiae* cells after encapsulation led to a reduction in Cd^2+^ biosorption sites [47]. However, encapsulation of yeast led to a more stable, easy-to-handle, economical, and scalable model than the use of free cells [20,25].

The Cd^2+^ biosorption of encapsulated *S. cerevisiae* at both tested concentrations was very fast, due to the abundance of binding sites [9]. At a contact time of 15 min, the encapsulated yeasts reached their maximum adsorption efficiency, and equilibrium was attained within 30 min (Figure 5). This implied that the alginate–yeast system displayed a good kinetic property for adsorbing Cd^2+^. At 30 min, the removal efficiency of Cd^2+^ was 51.1% ± 1.8% for aqueous solutions containing 5 mg/L Cd^2+^ and 51.2% ± 1.7% for those containing 10 mg/L Cd^2+^. This behavior remained without differences until 240 min at both tested concentrations (data not shown).

At a contact time of 15 min, the Cd^2+^ removal efficiency of empty Ca-alginate beads exposed to aqueous solutions containing 5 mg/L Cd^2+^ was higher (63.5% ± 0.7%) than the removal efficiency of the yeast–alginate system (51.1% ± 1.8%). However, the removal efficiency of empty Ca-alginate beads decreased gradually when the contact time increased (Figure 5), and their removal equilibrium was reached in 60 min when the removal efficiency was 46.2% ± 0.2%. At a contact time of 15 min, empty Ca-alginate beads exposed to aqueous solutions containing 10 mg/L Cd^2+^ showed a similar (*p* > 0.05) removal efficiency to that of the *S. cerevisiae*–alginate system. However, the removal efficiency of empty Ca-alginate beads decreased to 46.2% ± 0.2% when they reached equilibrium at 60 min.

**Figure 6:**
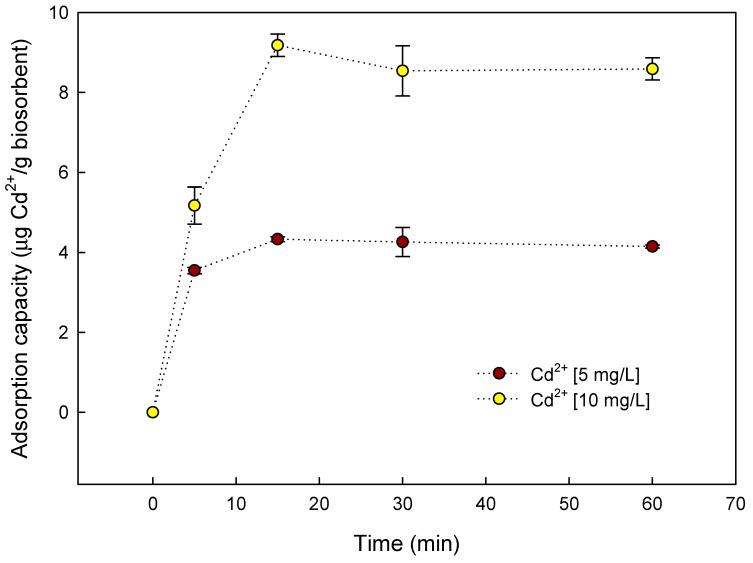
Effect of contact time on Cd^2+^ adsorption capacity of *S. cerevisiae*–alginate system at 25°C, pH 6.0, and different initial Cd^2+^ concentrations.

The biosorption capacities at 5 and 10 mg/L initial Cd^2+^ concentration were 4.3 and 9.2 µg of Cd^2+^/g of capsules (at 15 min of aqueous solution contact time), respectively (Figure 6). The probability of biosorption of Cd^2+^ on the active sites of the yeast–alginate system increased as Cd^2+^ became more concentrated in the aqueous solution, due to an increase in the driving force that overcame the mass transfer resistance of Cd^2+^ between the liquid and solid phases [48].

### 3.4. Fixed-Bed Column Biosorption

Fixed-bed column experiments were performed to investigate the potential application of the yeast–alginate system in a continuous process. During the column testing, a flow rate of 1.5 mL and Cd^2+^ solution resident time of 15 min were found to represent the best operational conditions. The total amount of Cd^2+^ (5 mg/L, pH 6.0) adsorbed by the yeast–alginate system was 185.2 µg and corresponded to a removal efficiency of 82.3% ± 2.3%. This percentage was higher than the removal efficiency of encapsulated *S. cerevisiae* in the batch study (51.9% ± 0.7%) using the same concentration and contact time, and even higher than the Cd^2+^ removal efficiency of free *S. cerevisiae* (75.0% ± 0.1%). A possible explanation for the increase in Cd^2+^ efficiency when changing from batch to continuous conditions is the increase in the driving force of Cd^2+^ due to the constant flux of solution [48].

The total adsorption capacity in fixed-bed columns was 15.4 µg of Cd^2+^/g of loaded yeast–alginate system beads, i.e., an increase of almost three-fold, with respect to batch experiments.

### 3.5. Desorption of Cd^2+^ from Fixed-Bed Column

Cadmium elution with 25% HNO_3_ allowed the recovery of 17.8% of total cadmium biosorbed by the *S. cerevisiae*–alginate system. In addition, a visible loss in the integrity of the capsules was observed after the contact with the eluent. The Cd^2+^ removal efficiency after HNO_3_ treatment was low compared with that reported by other authors (when using similar conditions to elute other toxic elements). For example, Ferraz et al. reported a Cr^3+^ recovery of 80% from a *S. cerevisiae* biosorbent system, while Chen and Wang observed a similar desorption efficiency for U^6+^ eluted from a yeast–alginate biosorbent system [49,50]. Stirk and Staden found that HNO_3_ was more effective for desorbing Cd^2+^ than other acids or bases [51]. However, several authors reported that the efficiency of nitric acid to elute Cd^2+^ from different biosorbents decreased significantly after a few cycles of regeneration due to the damage caused in the adsorbent [52]. Thus, it is necessary to continue testing different types of eluents to determine which is the most effective and economically appropriate.

### 3.6. FTIR Analysis

**Figure 7:**
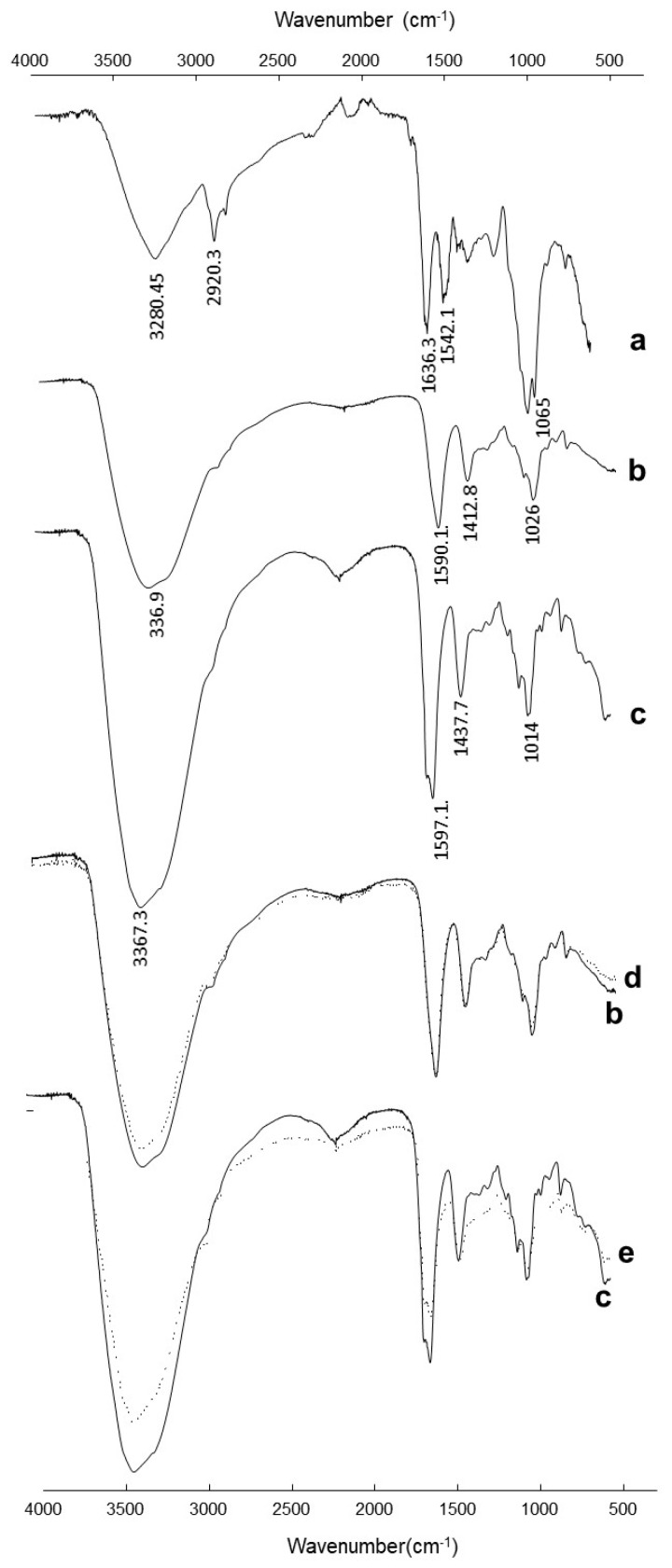
Fourier-transform infrared (FTIR) spectra of (**a**) free *Saccharomyces cerevisiae*, (**b**) empty Ca-alginate beads, (**c**) *Saccharomyces cerevisiae*–alginate system, (**d**) empty Ca-alginate beads after Cd^2+^ exposure, and (**e**) *Saccharomyces cerevisiae*–alginate system after Cd^2+^ exposure.

The spectrum of free *Saccharomyces cerevisiae* (Figure 7a) showed a broad adsorption band at 3000–3600 cm^−1^, representing –OH groups of carbohydrates and the –NH stretching of the protein. The bands observed at 2920.3 cm^−1^ and 2849.6 cm^−1^ were attributed to C–H stretching derived from acyl chains (–CH_2_ and –CH_3_). The bands at 1636.3 and 1542.1 cm^−1^ were attributed to amide I and amide II, respectively. Bands in the region from 1032.3 to 827.0 cm^−1^ were associated with carbohydrates, nucleic acids, and phosphate groups [53].

The spectrum of empty Ca-alginate beads showed all the characteristic peaks due to the alginate (Figure 7b). A broad band in the range of 3000–3600 cm^−1^ arose from the stretching of –OH groups. Very-low-intensity bands at 2919.3 cm^−1^ were attributed to stretching vibrations of aliphatic C–H. Observed bands between 1603.8 and 1415.8 cm^−1^ were attributed to asymmetric and symmetric stretching vibrations of COO^−^, respectively. The bands at 1100.0 and 935.0 cm^−1^ were attributed to the glycoside bonds in the alginate molecule (C–O–C stretching) [54]. The FTIR spectrum of the yeast–alginate system (Figure 7c) contained many signals characteristic for functional groups of both yeast and alginate, which indirectly confirmed the effective encapsulation of *Saccharomyces cerevisiae*.

FTIR analysis was used to characterize changes of functional groups before and after Cd^2+^ biosorption. Figure 7 shows the normalized spectra of the empty Ca-alginate beads before and after Cd^2+^ adsorption. The decrease in peak vibration intensity in the region between 3000 and 3600 cm^−1^ indicated that Cd^2+^ interacts with –OH formed in alginate beads. Hydroxyl groups were also designated as binding sites for Cu^2+^ and Zn^2+^ on the surface of alginate beads [55].

Changes in the intensity of 3367.3 to 3359.9 cm^−1^ in the Cd^2+^-loaded yeast–alginate system spectrum indicated that free –OH and –NH groups of immobilized *S. cerevisiae* probably bound with the metal.

In addition, alteration in wavenumbers at 2162.0, 1590.2, 1435.9, 1033.0, and 818.0 cm^−1^ could be assigned to the participation of aliphatic C–H, C=O asymmetric and symmetric stretching, phosphate groups, and C–O stretching from carbohydrates of yeast biomass groups in the biosorption process. Changes in the wavenumber intensity in the Cd^2+^-loaded yeast–alginate system (1598.1 cm^−1^) in comparison with the raw yeast–alginate system (1598.3 cm^−1^) emphasized the role of C=O stretching of *S. cerevisiae* carboxylates groups in metal uptake [56].

## 4. Conclusions

In this study, the Cd^2+^ biosorption properties of a yeast–alginate system were studied. A small gradual decrease in alginate gel strength over storage time was observed and attributed to an ion exchange process between calcium and some residual cations in solution, and also to a slow dissociation of calcium to aqueous media. However, the mechanical resistance of the yeast–alginate system was practically stable for 20 days. The FTIR attenuated total reflection (ATR) analysis of the yeast–alginate system before and after Cd^2+^ exposure showed that –OH formed in alginate beads and aliphatic C–H, C=O, phosphate groups, and C–O from carbohydrates of the yeast biomass groups participated in the biosorption process. The cadmium (Cd^2+^) removal efficiency and capacity using the *S. cerevisiae*–alginate system were higher in the fixed-bed column mode. This system allowed the removal of 83% of total Cd^2+^. Therefore, the *S. cerevisiae*–alginate system operated in the fixed-bed column mode has the potential to be used as an eco-friendly continuous water filter for Cd^2+^ biosorption.

## Figures and Tables

**Table 1:** Characterization of *Saccharomyces cerevisiae*–alginate system and empty Ca-alginate beads, before and after cadmium exposure.

Bead Type	Freshly Synthetized Bed Size (mm)	Moisture Content (%)	Surface Charge (mV)	After Lyophilization	
				Bead Size (mm)	Pore Size (µm)
Empty Ca-alginate	1.51 ± 0.02 ^a,^*	98.1 ± 0.1 ^a^	−14.3 ± 0.7 ^a^	1.49 ± 0.07 ^a^	0.66 ± 0.04 ^a^
Empty Ca-alginate exposed to Cd^2+^	N/D	N/D	7.5 ± 1.0 ^c^	0.89 ± 0.06 ^c^	0.28 ± 0.01 ^b^
*S. cerevisiae* encapsulated	1.62 ± 0.02 ^b^	96.0 ± 0.1 ^b^	−29.3 ± 1.5 ^b^	1.37 ± 0.05 ^a,b^	0.12 ± 0.01 ^c^
*S. cerevisiae* encapsulated exposed to Cd^2+^	N/D	N/D	2.7 ± 0.6 ^d^	1.15 ± 0.05 ^b,c^	0.10 ± 0.01 ^c^

* Values are expressed as means ± standard error. Different letters in the same column denote a statistical difference (*p* ≤ 0.05). N/D: not determined.
